# Etoposide Induces Nuclear Re-Localisation of AID

**DOI:** 10.1371/journal.pone.0082110

**Published:** 2013-12-04

**Authors:** Laurens J. Lambert, Simon Walker, Jack Feltham, Heather J. Lee, Wolf Reik, Jonathan Houseley

**Affiliations:** 1 Epigenetics Programme, The Babraham Institute, Cambridge, United Kingdom; 2 Centre for Trophoblast Research, University of Cambridge, Cambridge, United Kingdom; 3 Wellcome Trust Sanger Institute, Cambridge, United Kingdom; INSERM-Université Paris-Sud, France

## Abstract

During B cell activation, the DNA lesions that initiate somatic hypermutation and class switch recombination are introduced by activation-induced cytidine deaminase (AID). AID is a highly mutagenic protein that is maintained in the cytoplasm at steady state, however AID is shuttled across the nuclear membrane and the protein transiently present in the nucleus appears sufficient for targeted alteration of immunoglobulin loci. AID has been implicated in epigenetic reprogramming in primordial germ cells and cell fusions and in induced pluripotent stem cells (iPS cells), however AID expression in non-B cells is very low. We hypothesised that epigenetic reprogramming would require a pathway that instigates prolonged nuclear residence of AID. Here we show that AID is completely re-localised to the nucleus during drug withdrawal following etoposide treatment, in the period in which double strand breaks (DSBs) are repaired. Re-localisation occurs 2-6 hours after etoposide treatment, and AID remains in the nucleus for 10 or more hours, during which time cells remain live and motile. Re-localisation is cell-cycle dependent and is only observed in G2. Analysis of DSB dynamics shows that AID is re-localised in response to etoposide treatment, however re-localisation occurs substantially after DSB formation and the levels of re-localisation do not correlate with γH2AX levels. We conclude that DSB formation initiates a slow-acting pathway which allows stable long-term nuclear localisation of AID, and that such a pathway may enable AID-induced DNA demethylation during epigenetic reprogramming.

## Introduction

Genomes are protected from damage and mutation by a plethora of enzymes, however certain cell types perform carefully orchestrated DNA rearrangements and mutational programs that create or enhance population diversity. In B cells, VDJ recombination generates a naïve population of cells expressing different immunoglobulins (Ig). B cells are activated after encountering an antigen and proliferate while undergoing somatic hypermutation (SHM), a directed mutagenesis of the antigen binding region of the Ig that increases antigen affinity [1]. Some daughters of activated B cells also undergo class switch recombination (CSR), which changes the Ig constant region and alters downstream signalling in response to antigens [2]. 

The primary mutagen in both SHM and CSR is a single protein, Activation-induced cytidine deaminase (AID) [3,4], a member of the APOBEC family of RNA and DNA editing proteins that catalyse cytosine to uracil transitions (reviewed in 5). AID initiates CSR and SHM through subtly different mechanisms. In CSR, the uracil base formed by cytosine deamination is removed by uracil-DNA glycosylase (UNG), leaving an abasic site [6,7] at which the DNA backbone can be cleaved by apurinic endonuclease APE1 [8,9]. Multiple closely spaced cleavages occur in the CSR switch regions [10-12], forming staggered double strand breaks (DSBs) that can then be repaired by non-homologous end joining to yield the deleted CSR product (reviewed in 13). The UNG-mediated pathway also functions in SHM, which occurs in the context of rapid cell proliferation. Replication through an abasic site requires translesion synthesis with random replacement of the missing nucleotide, resulting in dC-dN mutations [14]. Mutations at dA:dT base pairs also occur in SHM although these cannot be directly introduced by AID/UNG. Instead, dU:dG mispairs produced by AID are recognised by the Msh2/Msh6 heterodimer [15-17], instigating a non-classical mismatch repair pathway that results in the re-synthesis of surrounding DNA by the error prone polymerase η [18].

AID has emerged as a candidate for epigenetic reprogramming as it has the potential to demethylate 5-methylcytosine (5mC). Direct deamination of 5mC by AID has been demonstrated *in vitro* [19], forming a dT:dG mismatch that could be repaired by thymine DNA glycosylase [20] and further processing to yield a demethylated dC:dG pair. Evidence also exists for the deamination of 5-hydroxymethylcytosine (5hmC) by AID [21]. However, recent studies have questioned this mechanism as AID prefers C to 5mC or 5hmC as a substrate *in vitro* [22-24], but AID could still demethylate 5mC indirectly by initiating homologous recombination or long patch repair at neighbouring residues [25,26]. Whatever the mechanism, compelling *in vivo* data links AID with epigenetic reprogramming: Aid^-/-^ mice show defects in the removal of DNA methylation during primordial germ cell (PGC) formation [27], and AID is required for the expression of key reprogramming factors during cell fusion reprogramming and iPS cell generation [28-30] and for the mesenchymal-epithelial transition in mammary epithelial cells [31]. AID can also demethylate DNA in early zebrafish embryos [32].

DNA deamination occurs in the nucleus, but though AID is technically small enough to diffuse through nuclear pores it is restricted to the cytoplasm and carries a specific nuclear import signal [33-35]. This import signal is offset by a strong Crm1-dependent nuclear export signal [34,35] and a cytoplasmic interaction with eEF1A that inhibits import [33,36]; as a result AID shuttles rapidly across the nuclear membrane with the vast majority remaining in the cytoplasm at steady state. To further limit activity, the stability of AID is low in the nucleus [37]; REG-γ targets AID for proteasomal degradation through an N-terminal motif [38] and a further destabilising motif is present at the C-terminal [39], although these negative regulators are partially offset by a stabilising interaction with YY1 [40].

Over-expressed AID is rigorously excluded from the nucleus, but enough protein transiently shuttles through the nucleus to cause detectable SHM [17], and SHM and CSR can be reconstituted by ectopic AID expression in NIH/3T3 cells showing that no B cell specific factors are required [41,42]. Furthermore, on-going SHM occurs in Ramos cells, a B cell lymphoma cell line that constitutively expresses AID without any sign of AID re-localisation to the nucleus [17,43,44]. These data suggest that no change in steady state AID localisation is required for function in B cells. Nonetheless, cells with nuclear AID are observed in germinal centres where B cell activation occurs [45] and also in sperm [46], suggesting that a dedicated pathway exists for AID nuclear re-localisation. Because other cell types express little if any AID relative to B cells, we speculated that any realistic role for AID in genome-wide demethylation would require a pathway to provide stable, long-term nuclear residence. Here we demonstrate that AID re-localises to cell nuclei for extended periods following DNA damage.

## Results

### Etoposide treatment causes nuclear AID re-localisation

**Figure 1:**
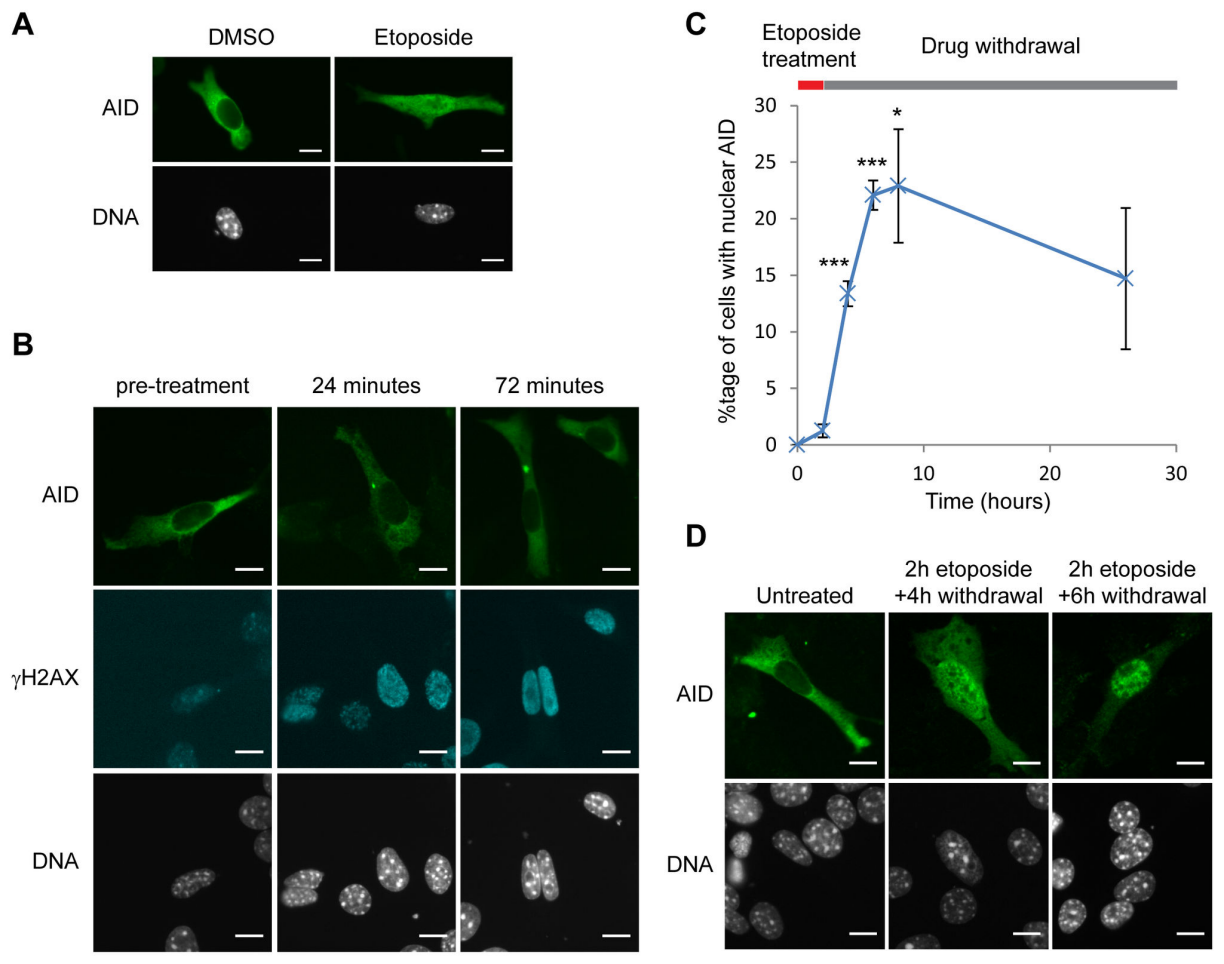
Etoposide induces AID re-localisation. A) AID localisation in NIH/3T3 cells transiently transfected with FLAG-AID and treated for 2 hours with 200µM etoposide or vehicle control (DMSO). Most etoposide-treated cells were indistinguishable from controls, however, the rare cells shown with nuclear AID (<1% transfected cells) in the drug treated sample were never observed in the controls. B) AID distribution after 24 and 72 minutes treatment with 200µM etoposide compared to γH2AX formation. Images were captured at equivalent exposure times to allow visual comparison of signal intensity. C) Percentage of transfected cells showing nuclear AID localisation after 2 hours treatment with 200µM etoposide followed by drug withdrawal for 0, 2, 4, 6 and 24 hours. Data is from three independent experiments, two using FLAG-AID and one using GFP-AID constructs to ensure that the specific tag was not the source of the effect. Error bars represent ±1 s.e., *** p<0.01, * p<0.05 for Student’s *t*-test comparing given time point to pre-treatment (time=0). D) AID localisation after 2 hours etoposide treatment followed by 4 and 6 hours drug withdrawal. Scale bars at bottom right of each image indicate 10µm.

**Figure 2:**
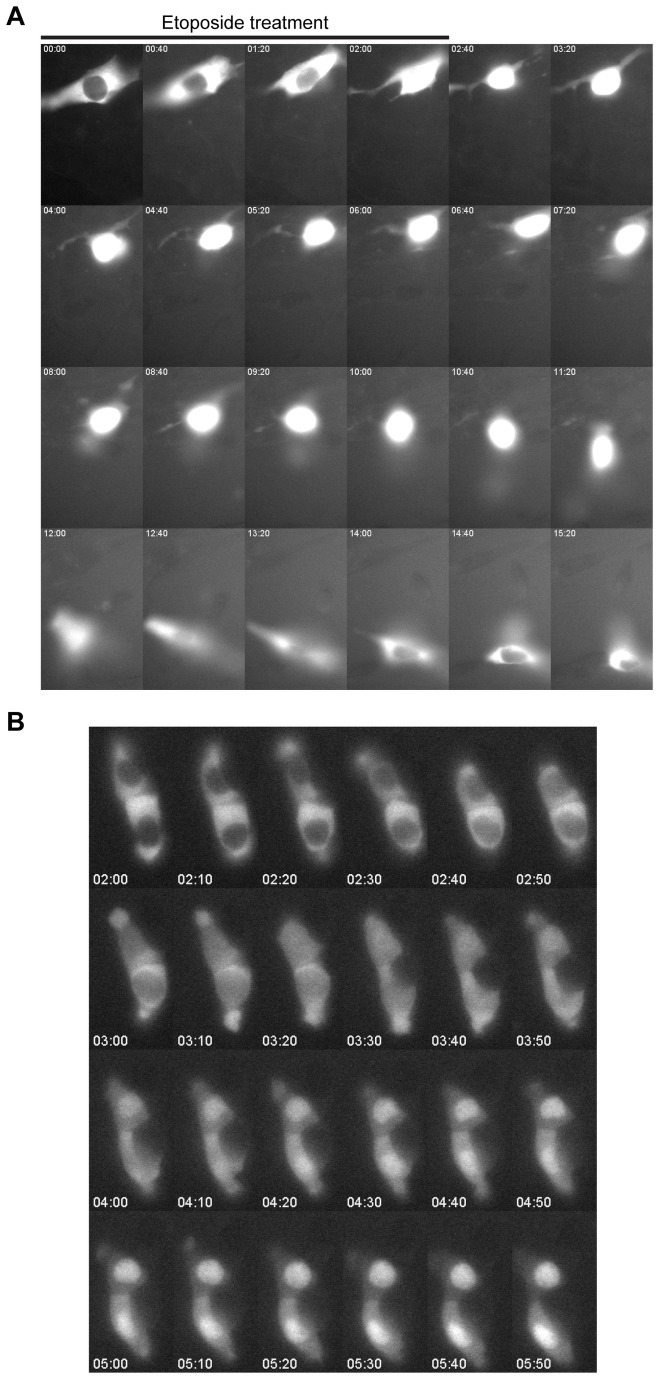
Dynamics of AID re-localisation. A) Montage of frames from live-cell imaging of an NIH/3T3 cell expressing GFP-AID, treated for 2 hours with 200µM etoposide and then monitored after drug withdrawal. Each frame represents 40 minutes. This video can be seen in full in Movie S1. B) Montage of two cells undergoing cytoplasmic to nuclear re-localisation of AID after etoposide withdrawal. Frames are shown at 10 minute intervals, t=0 at the addition of etoposide, first frame shown here is at etoposide removal. This video can be seen in full in Movie S3.

Nuclear accumulation of ectopically-expressed AID has been reported in HEK293 cells treated with γ-rays, hydrogen peroxide or bleomycin [47]. These agents induce DSBs, but only amongst complex patterns of DNA damage, leaving the connection between DSB formation and AID re-localisation uncertain (see 48 and references therein). We therefore examined AID localisation after treatment with etoposide, a suicide inhibitor of topoisomerase II that induces precise DSBs (reviewed in 49). A FLAG-AID construct was transiently transfected into NIH/3T3 cells and treated with etoposide for 2 hours. In untreated cells, AID localisation was exclusively cytoplasmic as expected from previous studies [17,41,42], but after etoposide treatment occasional cells were observed in which AID was present in the nucleus and cytoplasm (Figure 1A). We tested AID localisation after shorter etoposide exposures (24, 48, 72, 96 min), however, there was no increase in the number of cells showing nuclear AID despite the rapid appearance of H2AX phosphorylated at Ser139 (γH2AX) (Figure 1B). We then examined AID localisation during DSB repair by treating cells with etoposide for 2 hours, removing the drug and staining for AID after 2, 4, 6 and 24 hours. Across this time course, the proportion of cells showing detectable nuclear AID increased dramatically to a maximum of ~25% 4-6 hours after drug removal (Figure 1C). Greater re-localisation also occurred after drug removal, with many cells showing almost exclusively nuclear AID (Figure 1D). This phenotype was not caused by the presence of the N-terminal FLAG tag on the AID construct, as the same result was obtained with an N-terminal GFP tag (see later, Figure 2) and a C-terminal HA tag (Figure S1A). Therefore, AID re-localisation to the nucleus occurs in response to etoposide treatment, but is not a direct and immediate consequence of DSB formation.

### Dynamics of AID re-localisation

Etoposide is a potent cytotoxin and the concentration used in these experiments (200µM) is high enough to cause apoptosis, although no cell death was observed due to the short treatment time (2 hours). We were nonetheless concerned that the observed nuclear AID accumulation may be associated with cell death. We therefore used live-cell imaging to monitor NIH/3T3 cells expressing GFP-AID; immunofluorescence confirmed that GFP-AID and FLAG-AID responded similarly to etoposide (data not shown). As before, etoposide was applied for 2 hours followed by media change and cells were monitored for a further 14-19 hours after drug removal. Images were captured every ten minutes throughout drug treatment and after drug withdrawal, during which time cells remained live and motile. Although some cells disappeared during the timecourse, this was primarily due to cell movement out of the frame or plane of focus. In combination with the lack of evidence for dying cells or apoptotic phenotypes (such as nuclear blebbing) observed by DAPI staining, we conclude that a 2 hour treatment with 200µM etoposide does not cause cell death in NIH/3T3 cells in the short term.

Representative videos of AID re-localisation after etoposide treatment are presented in Movies S1-4. These experiments revealed that nuclear re-localisation of AID is not transient; about 35% of cells re-localised AID into the nucleus, on average 69 minutes after drug withdrawal though with a wide variation (standard deviation ±73 minutes for n=16 cells showing AID re-localisation, out of 42 cells imaged), measured as the time at which the nuclear signal exceeded the cytoplasmic signal. After re-localisation AID remained in the nucleus for an extended time (>10 hours), in most cases until the end of the experiment, although in three out of sixteen cases the protein returned to the cytoplasm (Figure 2A, Movie S1). Co-transfection with a nuclear-localised RFP construct confirmed the continuous nuclear localisation (Movie S5). The re-localisation of AID into the nucleus occurred over ~110 minutes (standard deviation ±25 minutes, n=16) (Figure 2B), and showed no further fluctuations across the extended nuclear residence. Of the 26 cells that did not undergo complete AID re-localisation, 24 showed no evidence of nuclear AID during the experiment suggesting that AID re-localisation is generally an all or nothing event. The two exceptions showed a very transient nuclear AID entry lasting 20-30 minutes (Figure S1B). These data show that after etoposide treatment, cells can stably maintain AID in the nucleus for extended periods (up to 19 hours) without undergoing cell death at least in the short term.

### Cell-cycle regulation of AID nuclear re-localisation

**Figure 3:**
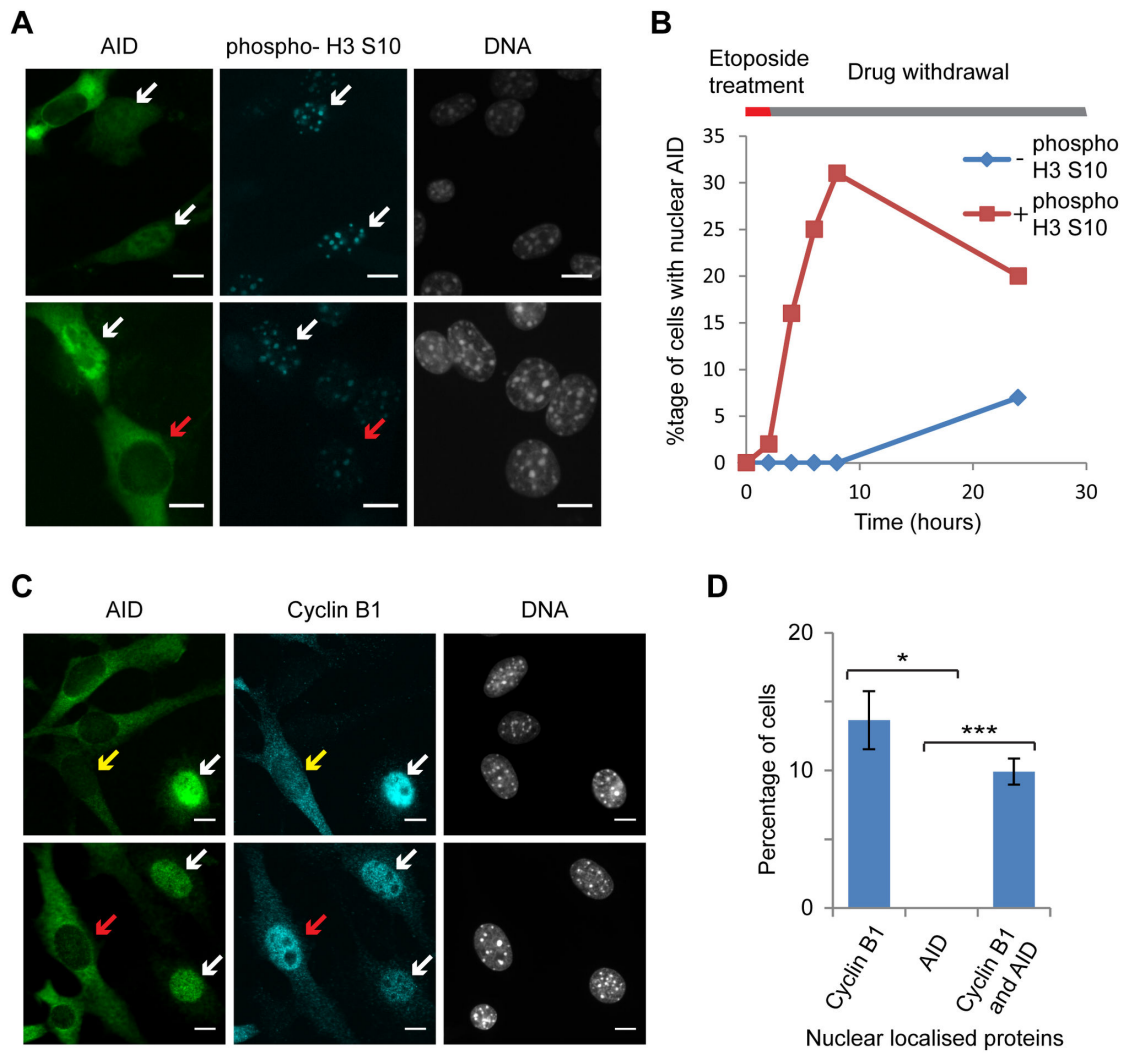
Cell cycle association of AID re-localisation. A) Co-staining of AID and phospho-H3 S10 in NIH/3T3 cells treated for 2 hours with 200µM etoposide, sampled 2 hours (upper panel) or 6 hours (lower panel) after drug withdrawal. White arrows indicate phospho-H3 S10 positive cells with nuclear AID, red arrows indicate a phospho-H3 S10 positive cell with cytoplasmic AID. B) Proportion of cells showing nuclear AID with and without phospho-H3 S10 across a time course of 2 hours 200µM etoposide treatment followed by drug withdrawal. C) Co-staining of AID and cyclin B1 in cells constitutively expressing FLAG-AID. Cells were treated with 200µM etoposide for 2 hours then allowed to recover for 5 hours before staining. White arrows indicate cells with nuclear AID and nuclear Cyclin B1, red arrows indicate a cell with cytoplasmic AID and nuclear cyclin B1, yellow arrows indicate a cell with cytoplasmic AID and cytoplasmic cyclin B1. D) Quantification of cells with nuclear cyclin B1, nuclear AID, or both. Error bars represent ±1 s.e., *** p<0.01, * p<0.05 for Student’s *t*-test, n=3 experiments with quantification of >100 cells each.

Nuclear-localised AID was only observed in a subset of treated cells, and etoposide produces DSBs primarily in S/G^2^ where topo II is most active [50], leading us to test the cell-cycle distribution of nuclear AID. Phosphorylation of histone 3 serine 10 (phospho-H3 S10) occurs initially at pericentric heterochromatin in G2 before dramatic upregulation at mitosis [51], and is therefore a good marker for G2 cells. Co-staining of phospho-H3 S10 during etoposide treatment revealed that all cells with nuclear AID were phospho-H3 S10 positive, consistent with re-localisation only in G2 (Figure 3A,B). The only exceptions were observed at 24 hours, most likely representing cells that had progressed through mitosis but maintained nuclear AID. 

To obtain better numerical data on AID re-localisation, we derived stable lines expressing the FLAG-AID construct. These 3T3-AID cells showed a similar etoposide response to transiently transfected cells, albeit with a lower percentage of cells showing AID re-localisation to the nucleus (10% compared to 25%). In these cells, we compared the nuclear localisation of AID to cyclin B1; cyclin B1 is expressed during G2 but is maintained in the cytoplasm by Crm1-dependent export until the start of mitosis [52,53]. 5 hours after etoposide withdrawal, AID nuclear localisation was only observed in cells with nuclear cyclin B1, never in cells with cytoplasmic or undetectable cyclin B1 (Figure 3C,D), refining the cell cycle stage of AID re-localisation to late G2/early prophase. 

### AID re-localisation is driven by DSB formation

In addition to inducing DSBs, etoposide increases supercoiling and chromosome catenation by inhibiting topo II, which may contribute to AID nuclear accumulation. To separate these effects, we compared etoposide to ICRF-193, a topo II inhibitor that induces minimal DSBs [54-56] and bleomycin, a radiomimetic that introduces DSBs through a non-topoisomerase based mechanism. We first assessed the dynamics of DSB induction by western blotting for γH2AX. As expected, ICRF-193 treated cells showed a minimal increase in γH2AX formation, whereas bleomycin and etoposide induced DSBs with similar dynamics; in both cases the γH2AX signal was maximal at the end of the 2 hour treatment and then slowly decreased after drug withdrawal (Figure S2A). However, treatment of cells expressing FLAG-AID with bleomycin or ICRF-193 (for 2 hours followed by 6 hours drug withdrawal) did not instigate detectable AID re-localisation (Figure S3B). This suggests that lack of topoisomerase II activity does not underlie the observed AID re-localisation, although compensation for loss of topoisomerase II function by topoisomerase I may be masking this effect.

**Figure 4:**
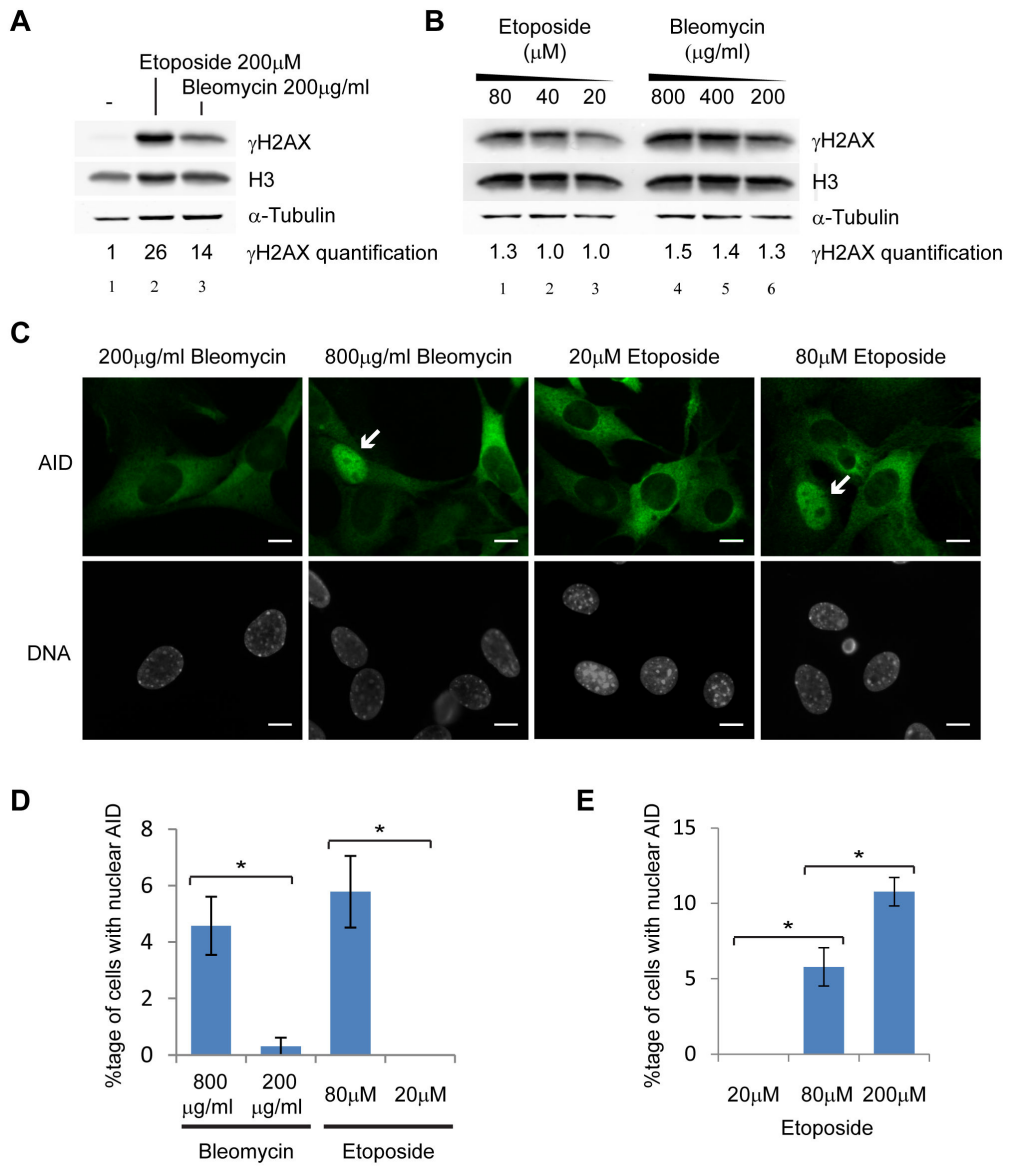
DSB requirement for AID nuclear localisation. A) Western blot showing γH2AX levels in NIH/3T3 cells treated for 2 hours with 200µM etoposide or 200µg/ml bleomycin, the concentration used in [47]. Histone H3 and α-tubulin are shown as loading controls, quantification of γH2AX is relative to tubulin. B) Western blot of γH2AX levels in NIH/3T3 cells after 2 hours treatment with given concentrations of etoposide and bleomycin. Controls as in A, quantification of γH2AX is relative to tubulin. C) Localisation of AID in a stable NIH/3T3 cell line expressing FLAG-AID, treated with bleomycin and etoposide at different doses for 2 hours and then allowed to recover for 5 hours. White arrows indicate cells with nuclear AID. Scale bars at bottom right of each image indicate 10µm. D) Quantification of cells shown in C with nuclear AID under different drug treatments. E) AID nuclear localisation with increasing etoposide concentration, cells treated as in C. Error bars represent ±1 s.e., * p<0.05 for Student’s *t*-test, n=3 samples per drug treatment except n=2 for 20µM etoposide and n=5 for 200µM etoposide, >100 cells were counted in each sample.

We noticed in these experiments that bleomycin treatment caused ~2-fold less H2AX phosphorylation than etoposide treatment (Figure 4A). We therefore treated cells with decreasing concentrations of etoposide (80, 40 and 20µM compared to 200µM used as standard) or increasing concentrations of bleomycin (800, 400 and 200µg/ml compared to 200µg/ml used as standard) for 2 hours. These concentration changes were sufficient to raise the γH2AX signal in bleomycin treated cells above that in etoposide treated cells (compare Figure 4B lanes 4 and 5 with 1-3), but the proportional changes in γH2AX levels across these four-fold concentration gradients were very slight (~1.3-fold, Figure 4B). We then treated 3T3-AID cells with the same concentrations of these drugs for 2 hours followed by 5 hours of drug withdrawal. In contrast to the slight γH2AX differences, the changes in AID nuclear localisation were dramatic: reduction of etoposide from 200 to 20µM almost abolished detectable nuclear AID re-localisation, while increasing bleomycin concentration from 200µg/ml to 800µg/ml caused 5% nuclear AID localisation (Figure 4C,D). 

The difference in AID localisation between 20μM and 80μM etoposide raised the possibility that AID nuclear re-localisation occurs only above a threshold drug concentration, which would have major implications for the mechanism. However, a significant increase in nuclear localisation was observed between 80μM and 200μM etoposide (Figure 4E), and very rare cells (<0.1%) with nuclear AID were detected in samples treated with 20μM etoposide (Figure S3). Therefore AID re-localisation is not a threshold effect, and the number of cells with nuclear AID increases with etoposide dose. Taken together, these data confirm that DSB formation, or another form of damage caused by both bleomycin and etoposide, instigates AID nuclear re-localisation, but underline the fact that this relationship is indirect.

## Discussion

Here we have re-examined the connection between DSB formation and AID nuclear accumulation. We confirm that DNA damaging agents are able to induce AID re-localisation, causing a complete and persistent re-localisation of AID from the cytoplasm to the nucleus. However, our data shows that this association is not direct; AID re-localisation occurs many hours after DSB formation (compare Figure 1C to Figure S2A) and does not obviously correlate to DSB level as marked by γH2AX. Particularly notable is the dramatic increase in nuclear AID localisation with increasing bleomycin concentration from 200 to 800µg/ml, or etoposide from 20µM to 80µM, despite a less than two-fold increase in γH2AX. We therefore conclude that AID accumulates in the nucleus in response to an as-yet unknown slow-acting signalling pathway that is triggered by these drug treatments. In addition to DSB formation, etoposide causes replication stress due to the collapse of replication forks colliding with the DNA-bound topoisomerase II legion, which may be the primary or an additional cause of AID re-localisation. We have not observed AID re-localisation after camptothecin treatment (data not shown), which should cause extensive replication fork collapse, however considering that drug concentration is critical for AID re-localisation in etoposide treated cells it remains possible that fork collapse is a contributing factor. Given the late-G2 dependence of the accumulation, it is also possible that the extended cell cycle arrest has a role in this, however, all tested concentrations of etoposide, bleomycin and ICRF-193 blocked entry into M-phase based on lack of condensed chromosomes, but did not necessarily cause AID re-localisation.

The proportion of cells showing AID nuclear re-localisation was highly reproducible between replicate experiments, but we noted that transient transfections show ~25% re-localisation whereas the stable cell line shows only ~10% for the same dose of etoposide. This may reflect a requirement for high levels of AID which would not be physiologically relevant, but may also reflect the physiology of the stable cell line; stable AID expressing lines are hard to derive, presumably due to AID-mediated mutagenesis, and may therefore survive by down-regulating AID import. 

The mechanism that maintains cytoplasmic AID has been well-characterised, and strong AID nuclear accumulation phenotypes have been observed after removal of the Crm1-dependent nuclear export signal or inhibition of Crm1 [34,35]. One explanation for etoposide-mediated AID re-localisation may be that etoposide causes a general inhibition of Crm1. Alternatively, the C terminal NES of AID may be modified (as occurs for Cyclin B1) or masked by a protein, preventing interaction with Crm1, although we know it is not cleaved as the anti-AID mAb used in this study binds to the NES. Blocking or modification of the NES may be relevant to AID function in our system. Given the mutagenicity of AID, it is certainly surprising that cells can tolerate high nuclear concentrations for many hours; the DSB forming CSR-type activity is likely to be particularly damaging. However, mutations in the C-terminal NES of AID can block CSR while leaving SHM functional [39,57-59], so if nuclear retention is mediated by masking or modifying the NES region, this modified AID may not actually produce widespread chromosome breakage. Nonetheless the mutagenicity of nuclear-restricted AID is higher than wild-type so significant mutagenesis would still be expected [34,57,60].

While genetic studies have strongly implicated AID in DNA demethylation, these are at odds with known biochemical properties of AID. One major issue is that for SHM or CSR, only a small amount of protein needs to be accurately targeted to a few sites in the genome. This is feasible with the small amount of AID that transiently shuttles into the nucleus. Genome-wide demethylation in contrast would require a large amount of protein and/or a very long time to cover the whole genome. Given that PGCs and iPS precursor cells express far less AID than B cells, efficient nuclear exclusion of AID would prevent useful amounts of AID remaining in the nucleus for sufficiently long to perform meaningful demethylation. Our data suggests the first resolution to this issue by demonstrating the existence of a pathway that completely re-localises wild type AID into the nucleus where it remains for many hours.

## Materials and Methods

### Cell culture and drug treatment

NIH/3T3 cells were cultured at 37°C, 5% CO_2_ in DMEM (Life Technologies 41966-052) + 10% calf serum. Etoposide (Sigma) was added to cultures at 200µM except where specified otherwise from a 50mM stock in DMSO, DMSO was used as a vehicle control. Other drugs were used at stated concentrations: Bleomycin (Santa Cruz), stock 10mg/ml in water, and ICRF-193 (Santa Cruz), stock 10mM in DMSO. 

### Plasmids and transfections

FLAG-AID (pRH125-AID) and AID-HA (pMX-AID-HA) were gifts from C. Rada. For GFP-AID, AID was amplified from FLAG-AID with oligos AGTCTGCTAGCTCGAGCTATGGACAGCCTCTTGATGAAC and GATCGATCGTGGATCCTATCAAAGTCCCAAAGTACGAA, and cloned into pEGFP-C1 using *Bam*HI and *Xho*I. pNLS-Cherry was a gift from L. Roderick. Transfections were performed with FuGENE 6 (Promega) according to manufacturer’s instructions, stable lines cells were selected with 7.5µg/ml puromycin (Sigma).

### Immunofluorescence

Cells were grown directly on glass coverslips, in some cases coated with poly-L-lysine, and fixed with 4% formaldehyde in PBS for 10 min. After washing with PBS and permeabilising with 0.5% Triton-X100 in PBS for 10 min, cells were treated with blocking solution (5% ELISA blocking solution Roche 11112589001 in PBS) for 30 min at room temperature, followed by staining with primary and secondary antibodies in blocking solution each for 30 min at room temperature. Alternatively, for HA and cyclin B1 staining, after fixation and washing cells were blocked for 1 hour in 5% goat serum, 0.3% Triton-X100 in PBS, then incubated overnight at 4°C with the primary antibody in 1% BSA, 0.3% Triton-X100 in PBS. After washing with PBS, cells were stained for 1 hour with the secondary antibody in the same buffer. Slides were dehydrated through 70%, 90%, 100% ethanol before mounting in Vectashield with DAPI. Primary antibodies: rat α-AID (EK2 5G9 [46], which binds a motif at the C-terminal of AID) at 1:50, rabbit α-γH2AX (CST 9718) at 1:400, rabbit α-phospho-H3S10 (Santa Cruz sc-8656), rabbit α-GFP (Life Technologies A11122) at 1:500, rabbit α-cyclin B1 (CST 12231) at 1:200, mouse anti-HA (CST 2367) at 1:100. Secondary antibodies were AlexaFluor 488 and 594-conjugates from Molecular Probes (Life Technologies) used at 1:1,000.

### Microscopy

Immunofluorescence images were captured using an Olympus BX-41 system with a 40x oil immersion objective. Live cell imaging was performed on an Olympus Cell^R imaging system comprising an inverted IX81 microscope, 40x 1.3 NA oil immersion objective, Polychrome V illumination system (Till Photonics) and ORCA-ER CCD camera (Hamamatsu). Cells were imaged for GFP (ex 488/10, em 525/40) and mCherry (ex 585/10, em 640/75) as required. Cells were maintained in a 5% CO_2_ atmosphere, and were treated with 200 µM etoposide in standard media for 2 hours. After drug treatment, media was flushed several times and cells then maintained in standard media. Cells were typically followed for 18-20 hours with images captured every 10 minutes with the imaging system controlled using Olympus SIS software. All images were processed for publication using ImageJ, images were cropped and merged as required, and maximum and minimum signal levels adjusted linearly.

### Protein isolation and western blotting

Cells covering 4cm^2^ (1 well of a 12-well plate) were scraped in ice-cold PBS and pelleted at 300g for 1min before re-suspension in 100µl ice cold RIPA buffer + Complete Protease Inhibitors (Roche). Cells were incubated on ice for 15min then sonicated 4x20s in a Bioruptor set to High, before centrifuging for 10min at 10,000g. 20µl lysate was loaded per well on 5-12% Tris-glycine gels and transferred to nitrocellulose membrane. Antibody staining was performed using standard methods for fluorescence detection (see protocols at www.cellsignal.com) and imaged using a LI-COR Odyssey system. Antibodies were rabbit α-γH2AX (CST 9718) at 1:1,000, rabbit α-H3 (Millipore 07-690) at 1:5,000 and mouse α-tubulin (CST 3873) at 1:10,000, DyLight-conjugated secondary antibodies (CST) were used at 1:15,000.

## Supporting Information

Figure S1 **Nuclear re-localisation of AID.** A) Nuclear re-localisation of AID in NIH/3T3 cells transiently transfected with an AID-HA construct. Cells were treated with 200µM etoposide followed by 6 hours of drug withdrawal before staining. No nuclear localised AID was observed amongst untreated control cells. B) Montage of frames from live-cell imaging of an NIH/3T3 cell expressing GFP-AID, treated for 2 hours with etoposide followed by drug withdrawal. Each frame represents 10 minutes. This is one of only two cells observed (out of 42) that underwent transient nuclear re-localisation of AID. This video can be seen in full in Movie S4.
(TIF)

Figure S2 **Dynamics of γH2AX compared to AID localisation.** A) Western blots showing γH2AX accumulation in NIH/3T3 cells treated with no drug, etoposide (200µM), bleomycin (200µg/ml) or ICRF-193 (100µM), samples were taken across a 2 hour drug treatment and 6 hours of drug withdrawal. H3 is shown as a loading control. B) Localisation of AID in a stable NIH/3T3 cell line expressing FLAG-AID, treated with etoposide, bleomycin or ICRF-193 doses given in A for 2 hours followed by 6 hours of drug withdrawal. Nuclear AID was only observed in etoposide-treated cells.
(TIF)

Figure S3 **AID re-localisation at low etoposide concentration.** Cells from Figure 4C treated with 20µM etoposide showing clear nuclear AID re-localisation. These cells were extremely rare (only a few were seen per cover slip), and were not encountered while performing the cell counts for Figure 4C. However, such cells were never observed in untreated samples.
(TIF)

Movie S1 **AID re-localisation in response to etoposide treatment.** NIH/3T3 cells were transiently transfected with GFP-AID, treated with 200µM etoposide for 2 hours then monitored after drug withdrawal. Video starts at the application of etoposide and images were captured every 10 minutes.
(AVI)

Movie S2 **AID re-localisation in response to etoposide treatment.** Movie of GFP-AID re-localisation in response to etoposide treatment, as Movie S1.
(AVI)

Movie S3 **AID re-localisation in response to etoposide treatment.** Movie of GFP-AID re-localisation in response to etoposide treatment, as Movie S1.
(AVI)

Movie S4 **AID re-localisation in response to etoposide treatment.** Movie of GFP-AID re-localisation in response to etoposide treatment, as Movie S1.
(AVI)

Movie S5 **AID re-localisation relative to nucleus.** As Movie S1, but cells were co-transfected with a nuclear-localised RFP construct as a nuclear marker. AID is shown in green, RFP in red.
(AVI)
