# Supplementary figures and images for: Etoposide Induces Nuclear Re-Localisation of AID
Source: PLoS One. 2013 Dec 4;8(12):e82110. doi: 10.1371/journal.pone.0082110 (PMC3852760; doi:10.1371/journal.pone.0082110)

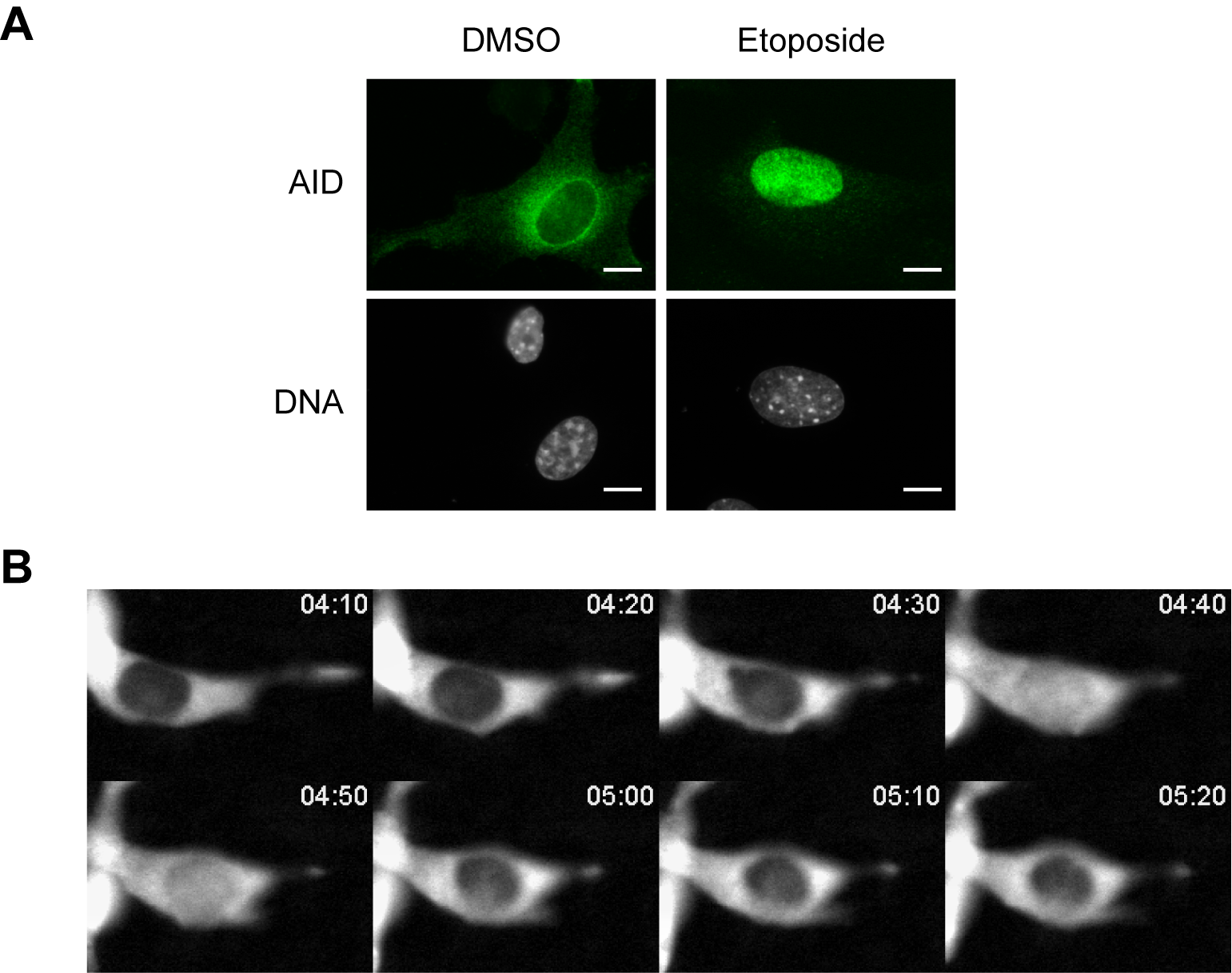

Supplement: Figure S1 — Nuclear re-localisation of AID. A) Nuclear re-localisation of AID in NIH/3T3 cells transiently transfected with an AID-HA construct. Cells were treated with 200µM etoposide followed by 6 hours of drug withdrawal before staining. No nuclear localised AID was observed amongst untreated control cells. B) Montage of frames from live-cell imaging of an NIH/3T3 cell expressing GFP-AID, treated for 2 hours with etoposide followed by drug withdrawal. Each frame represents 10 minutes. This is one of only two cells observed (out of 42) that underwent transient nuclear re-localisation of AID. This video can be seen in full in Movie S4. (TIF) [file pone.0082110.s001.tif]

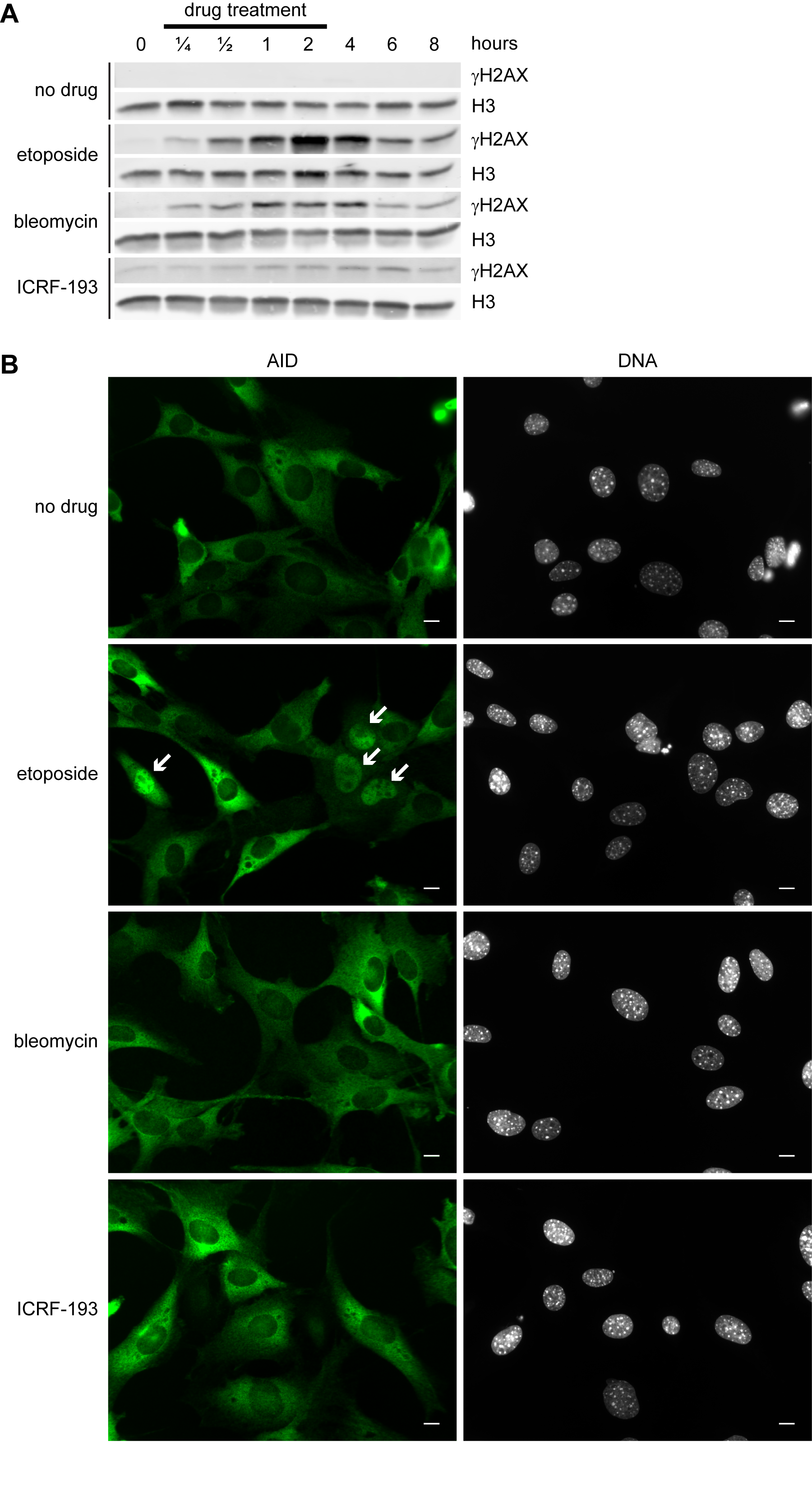

Supplement: Figure S2 — Dynamics of γH2AX compared to AID localisation. A) Western blots showing γH2AX accumulation in NIH/3T3 cells treated with no drug, etoposide (200µM), bleomycin (200µg/ml) or ICRF-193 (100µM), samples were taken across a 2 hour drug treatment and 6 hours of drug withdrawal. H3 is shown as a loading control. B) Localisation of AID in a stable NIH/3T3 cell line expressing FLAG-AID, treated with etoposide, bleomycin or ICRF-193 doses given in A for 2 hours followed by 6 hours of drug withdrawal. Nuclear AID was only observed in etoposide-treated cells. (TIF) [file pone.0082110.s002.tif]

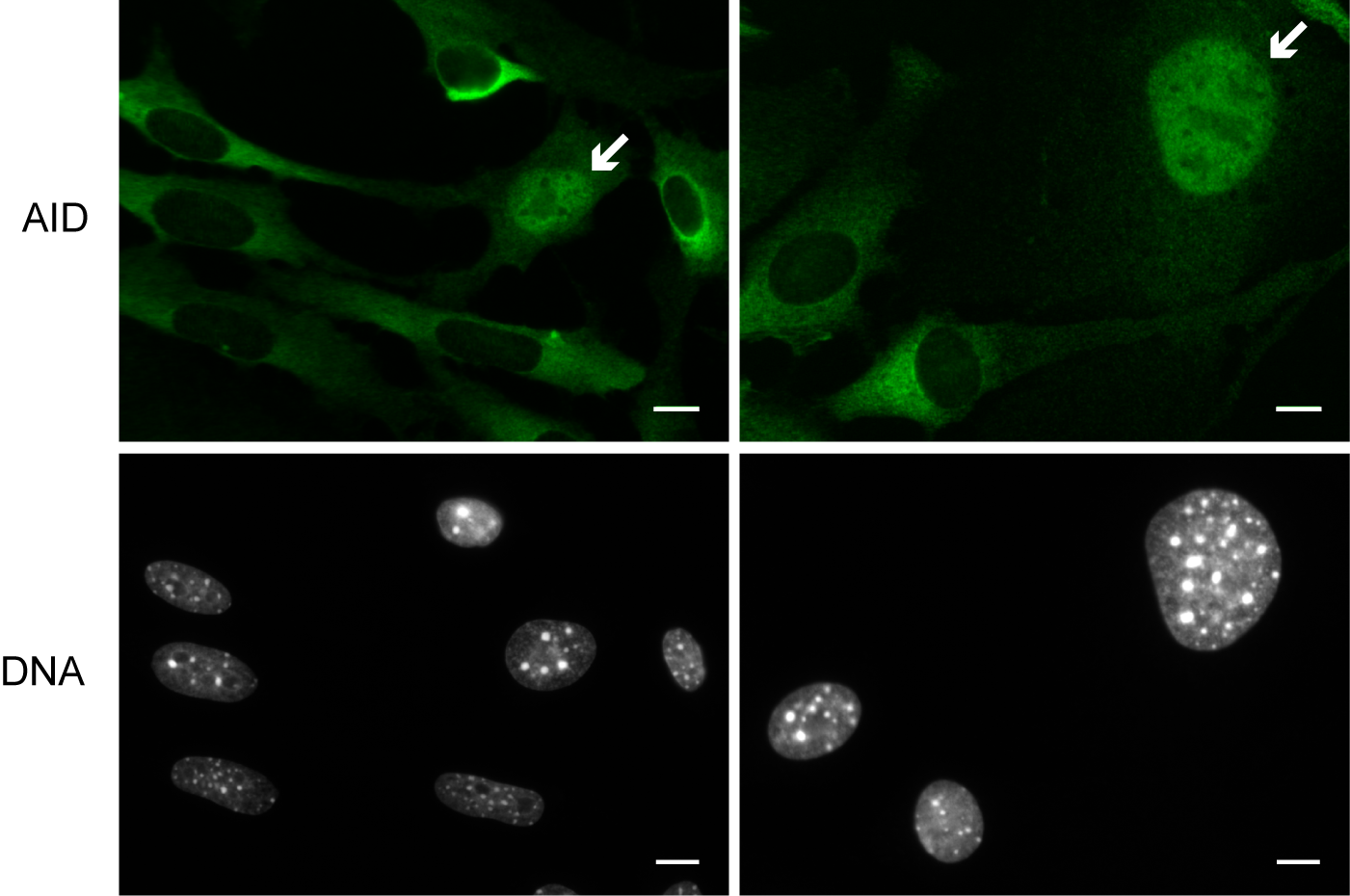

Supplement: Figure S3 — AID re-localisation at low etoposide concentration. Cells from Figure 4C treated with 20µM etoposide showing clear nuclear AID re-localisation. These cells were extremely rare (only a few were seen per cover slip), and were not encountered while performing the cell counts for Figure 4C. However, such cells were never observed in untreated samples. (TIF) [file pone.0082110.s003.tif]
